# Nitrogen Cycling in a Norway Spruce Plantation in Denmark — A SOILN Model Application Including Organic N Uptake

**DOI:** 10.1100/tsw.2001.394

**Published:** 2001-12-12

**Authors:** Claus Beier, Henrik Eckersten, Per Gundersen

**Affiliations:** RISO National Laboratory, Roskilde, Denmark

**Keywords:** dynamic modelling, mycorrhiza, nitrogen addition, nitrogen cycling, organic nitrogen uptake, spruce forest

## Abstract

A dynamic carbon (C) and nitrogen (N) circulation model, SOILN, was applied and tested on 7 years of control data and 3 years of manipulation data from an experiment involving monthly N addition in a Norway spruce ( Picea abies, L. Karst) forest in Denmark. The model includes two pathways for N uptake: (1) as mineral N after mineralisation of organic N, or (2) directly from soil organic matter as amino acids proposed to mimic N uptake by mycorrhiza. The model was parameterised and applied to the data from the control plot both with and without the organic N uptake included. After calibration, the models performance was tested against data from the N-addition experiment by comparing model output with measurements. The model reproduced well the overall trends in C and N pools and the N concentrations in soil solutions in the top soil layers whereas discrepancies in soil-solution concentrations in the deeper soil layers are seen. In the control data, the needle-N concentration was well reproduced except for small underestimations in some years because of drought effects not included in the model. In the N-addition experiment, SOILN reproduces the observed changes; in particular, the changes in needle-N concentrations and the overall distribution within the ecosystem of the extra added 3.5 g N m(-2) year(-1) parallel the observations. When organic N uptake is included, the simulations indicate that in the control plot receiving c. 1.9 g N m(-2) year(-1), the organic N uptake in average supplies 35% of the total plant N uptake. By addition of an extra 35 kg N ha(-1) year(-1), the organic N uptake is reduced to 16% of the total N uptake. Generally, inclusion of the pathway for organic N uptake improves model performance compared with observations for both C and N. This is because mineral N uptake alone implies a larger mineralisation rate, leading to bigger concentrations of N in the soil and soil water, bigger N losses, and net loss of c. 100 kg C ha(-1) year(-1), thereby causing depletion of the organic soil layer.

### INTRODUCTION

Nitrogen (N) has, in many cases, been a growth-limiting factor in European forests[1]. During the last 50 years, however, increased anthropogenic emissions of N have also increased N availability. It has been hypothesised that this may be one of the main reasons for increased forest growth observed in some areas during the same period[2]. Despite that, N supply from the atmosphere may now exceed the demand for forest growth in many areas of Europe. N-saturated conditions may have occurred as indicated by elevated N leaching from forest ecosystems[3]. Nitrate leaching may cause soil acidification, accelerate base cation losses from the soil, and increase the input of N and base cations to downstream water environments.

A number of ecosystem studies have been initiated over the last decade to gain insight into N dynamics and especially the impact of increased N load on forest ecosystems[4,5,6]. In order to generalise the results from these ecosystem studies and to predict the impact of future atmospheric N loads, interest in mathematical models focusing on N dynamics has increased[7,8]. SOILN[9,10,11] is such a model of C and N flows in soil and plants based on a current understanding of the process. In this article, we apply SOILN to a plantation of Norway spruce (*Picea abies*, L. Karst) at Klosterhede, Denmark where a N-addition experiment was carried out[12]. SOILN was first applied by the assumption that N is mineralised to its inorganic forms before plants can take it up. This led to an unrealistic high loss of C from the soil because the mineralisation rate required satisfying the N uptake was too high compared to the observation. Recent findings have shown that N can be transferred directly as amino acids from the organic pool to the plants, preferably as a result of mycorrhizal symbioses with the plants[13,14,15,16], which may be an important pathway in nutrient-poor forest ecosystems[17]. SOILN was therefore reformulated to allow uptake of organic N from soil organic matter and this version was applied to the site as well.

The main objectives of the study were to test the SOILN-model predicting the N and C balances of forests against a range of observed parameters from an old spruce stand and to compare the C and N dynamics in model formulations with and without organic N uptake.

## EXPERIMENTAL METHODS AND MODEL DESCRIPTION

### Site and Treatments

The study site was a 76-year-old (1995) Norway spruce plantation located at Klosterhede, Lemvig in western Jutland (8°24′E, 56°29′N), Denmark. The stand is the second-forest generation after heathland afforested 120 years ago. The soil is classified as a Typic Haplorthod (podzol) developed on a homogeneous sandy, nutrient-poor deposit of a glacio-fluvial outwash plain. The soil is coarse sand with low clay content (<4%). An organic layer (mor humus) of c. 7 cm has developed during the current rotation. Throughfall and soil water chemistry is dominated by high sea-salt input from the North Sea 15 km away. The total deposition of N was approximately 2 g N $m^{-2}$ $year^{-1}$ measured in throughfall. The Klosterhede site is situated in a region that still responds to N fertilisation, and at the site water is known to play a significant role in regulating tree growth[18]. Therefore, the stand may be only partly limited by N.

An untreated control plot was established in 1983 and two additional plots in 1990. A N-addition experiment was carried out next to the control plots in the period from 1992 to 1996 by application of 3.5 g N $m^{-2}$ $year^{-1}$ ($NH_4NO_3$) to simulate a total input of 5.0–5.5 g N $m^{-2}$ $year^{-1}$. All plots were c. 500 $m^2$ and all measurements were performed within the central 15 × 15 m. To trace the fate of the N deposition and the added N, labelled $^{15}N$ (0.11 g N $m^{-2}$ $year^{-1}$ of a 30% enriched $^{15}NH_4{}^{15}NO_3$ salt) was added to the N-addition plot and one of the control plots. A more detailed site description is given elsewhere[18,19] as well details on the experimental design and treatment results[12,20].

## MEASUREMENTS AND DATA PREPARATION

### Input and Output of N

The input of N to the site was measured monthly by collection of bulk precipitation and throughfall in funnels. The mean inputs of $NO_3$ and $NH_4$ (1989 to 1995) in bulk precipitation are 0.24 and 0.27 g N $m^{-2}$ $year^{-1}$, respectively, and 1.06 and 1.15 g N $m^{-2}$ $year^{-1}$, respectively, in throughfall[21]; litterfall fluxes averages 2.7 g N $m^{-2}$ $year^{-1}$[12]. The average input of water by precipitation is 893 mm $year^{-1}$ and the estimated average percolation of water is 356 mm $year^{-1}$; leaching of N is negligible (<0.01 g N $m^{-2}$ $year^{-1}$)[21].

### Soil C and N Pools

The total accumulation of organic C and N in the soil profile was determined by soil coring. Separation of the organic C and N pools into litter, humus, and microbial pools was roughly estimated according to two criteria:

- The easily decomposable fraction of soil organic matter (litter) in the top soil layer is set to 4 years of above-ground litterfall. With a 50% weight loss per year[19] for the fresh and easily decomposable organic material, it can be calculated that the litter pool at steady state will asymptotically approach two times the yearly litter input. If above- and below-ground litter production is assumed to be equal, the total litter-pool equals 4 years of above-ground litterfall.
- The ratio of litter/humus should be highest in the top soil and gradually decrease in the deeper soil layers.

Finally, the separation of the organic C and N pools into litter, humus, and microbial pools was estimated using the model. Making a simulation over several years using the current parameter settings. The simulated average litter and microbial pools stabilised at certain fractions of the total organic pool. These fractions were used to separate the observed total organic matter into litter, humus, and microbial biomass, in each layer, respectively (Table 1).

### Biomass and Above-Ground C and N Pools

The above-ground biomass was estimated at the end of the experiment by felling 12 trees representing the various size classes at the site. The biomass pools of wood, bark, branches, and needles were measured and empirically related to the individual tree diameter at breast height (DBH). The equations were compared to corresponding equations developed for Sweden[22] showing a good agreement. The yearly changes in the above-ground

**TABLE 1**
**Total Measured C and N Pools in the Soil and Estimated Distributions into Fractions of Soil Organic Matter and Soil Layers**

| | Layer and Depth | | | | | | |
|---|---|---|---|---|---|---|---|
| | **1 0–7 cm** | **2 8–15 cm** | **3 16–17 cm** | **4 18–20 cm** | **5 20–55 cm** | **6 55–80 cm** | **Total Profile** |
| | | | | $10^3$ g C $m^{-2}$ | | | |
| Microbial C | 0.2 | 0.05 | 0.007 | 0.007 | 0.017 | 0.008 | 0.29 |
| Litter C | 0.4 | 0.08 | 0.01 | 0.01 | 0.03 | 0.006 | 0.54 |
| Humus C | 11.0 | 2.72 | 0.49 | 0.98 | 3.43 | 0.63 | 19.4 |
| **Total C** | **11.6** | **2.85** | **0.51** | **1.00** | **3.45** | **0.64** | **20.1** |
| | | | | mg C $cm^{-3}$ | | | |
| Density | 162 | 35 | 25 | 33 | 10 | 2.6 | |
| | | | | g N $m^{-2}$ | | | |
| Microbial N | 12.5 | 2.8 | 0.4 | 0.4 | 0.9 | 0.4 | 17.4 |
| Litter N | 12 | 2.4 | 0.32 | 0.32 | 0.9 | 0.16 | 16.1 |
| Humus N | 363 | 87 | 16 | 32 | 111 | 20 | 629 |
| **Total N** | **388** | **92** | **16.7** | **32.7** | **113** | **21.5** | **663** |
| | | | | mg N $cm^{-3}$ | | | |
| Density | 5.4 | 1.1 | 0.8 | 0.1 | 0.03 | 0.01 | |

*Note:* The organic soil layer is 0 to 7 cm.

biomass pools were calculated from measurements of DBH and the estimated relationships, while root biomass was calculated by the relationship for Norway spruce in Sweden[22]. The N and C concentrations in the various biomass fractions were measured each year in October and used to calculate the pools of C and N (Table 2.)

### Model Description – SOILN

The SOILN model is a field-scale model simulating one-dimensional C and N flows in soil and plant driven by daily inputs of air temperature and solar radiation, and soil heat and water conditions. The plant compartment assimilates C from the atmosphere ($W_t'$) proportional (e) to the intercepted solar energy ($I_{Intercepted}$) [23]:

$$W_t' = f_T f_W f_N e I_{Intercepted} \quad (1)$$

where $f_T$, $f_W$, and $f_N$ are linear-response functions of growth to daily air temperature, transpiration to potential transpiration ratio, and needle N concentration, respectively. The functions range between 0 and 1, and parameter values are taken from other stud-

**TABLE 2**
**N and C Content and Pool Sizes in the Biomass used in the SOILN Calibration**

| Compartment | Biomass (g $m^{-2}$) | N-conc. (%) | N (g $m^{-2}$) | C-conc. (%) | C (g $m^{-2}$) |
|---|---|---|---|---|---|
| Needles | 1500 | 1.2–1.5 | 20 | 51 | 800 |
| Branches | 3100 | 0.7 | 21 | 52 | 1600 |
| Stems | 15,400 | 0.15 | 23 | 49 | 7500 |
| **Total Above** | **20,000** | | **64** | | **9900** |
| | | | | | |
| Stump | 1400 | 0.15 | 2 | 49 | 700 |
| Fine roots | 1000 | 0.7 | 7 | 52 | 500 |
| Big roots | 3000 | 0.7 | 27 | 52 | 1600 |
| **Total below** | **5400** | | **36** | | **2800** |

ies[10]. Growth is assumed to start at +2°C and needle-N concentrations above 0.5%. Growth reaches maximum at 10°C and needle-N concentrations of 2%[24]. Allocation of growth to needles, stems (woody parts), and roots follows allometric coefficients of which the fraction allocated to roots decreases at low needle-N concentrations[25]. N is taken up from soil and distributed in plants in accordance with growth of different tissues and their N demand (see $N_{Demand}$ below). Litterfall is a fraction of the standing biomass, consisting of needles and woody parts. The litterfall on the ground surface and root litter is distributed in the soil profile. C/N ratio of litter equals the source except for needles, where 20% of N is redrawn before abscission.

The soil compartment is divided into horizontal layers. In each layer, C and N can be stored in organic matter represented by three pools: easily decomposed organic material (“litter”), slowly decomposed organic matter (“humus”), and microbial biomass. Litterfall of both needles and roots enters the litter pool before uptake or transfer to other pools. Decomposition of the organic matter follows different routes (Fig. 1). A fraction of the microbial C assimilation is transferred directly to the humus pool as humification, a fraction of both litter and humus can be transferred directly as amino acids from the organic pools to the plants (see below) and, finally, organic matter is decomposed at decomposition rates specified for each pool. N from deposition, fertilisation, and mineralisation enters the mineral N pool represented by one pool for ammonium N and one for nitrate N. Nitrate is fully transportable with the soil water. Only a fraction of the ammonium is mobile because ammonium can be adsorbed in the soil modelled by sorption isotherms[26]. The soil processes represented are plant N uptake, microbial growth and N mineralisation, nitrification, denitrification, and vertical transport of N with water.

Root N uptake is the lowest value of plant demand for N ($N_{Demand}$) and the amount available for root uptake ($N_{Available}$)[27].

$$N_{Soil \rightarrow Plant} = \min\,(N_{Demand}, N_{Available}) \quad (2)$$

The plant N demand equals the potential uptake, defined as the daily growth multiplied by the maximum N concentration of tissues; needles, stems, and roots are treated separately. N availability is the sum of soil mineral N possible to take up by roots ($c_u N_{Mineral}$)[9] and the ability to take up organic N[28]

$$N_{Available} = c_u N_{Mineral} + f_{Def}(c_{Li} N_{Litter} + c_h N_{Humus}) \quad (3)$$

where $c_u$, $c_{Li}$, and $c_h$ are the coefficients for the efficiency of plant N uptake from the mineral pool and the two organic pools. Uptake of N directly from the organic pools is assumed to imitate a hypothesised N transfer from the organic N pool to the plants facilitated by mycorrhizal fungi. It is assumed that this organic N transfer directly from the litter and humus pools to the roots occurs in the form of amino acids with a C/N ratio of 4. Hence the mycorrhizal fungi is assumed to be a part of the root system and the low C/N ratio tends to increase the N concentration of the root system. The uptake from the organic pools occurs only if the mineral uptake does not meet the plant N demand, i.e., there is an excess of C in the growing tissues. The deficiency factor ($f_{Def}$ ) is expressed as the ratio between actual deficiency of mineral N supply and a threshold deficiency ($c_{Def}$) above which the uptake is only limited by the availability of organic N.

$$f_{Def} = (N_{Demand} - c_u N_{Minera})/c_{Def}\,; \quad < 1 \quad (4)$$

The N dynamics of microbes are driven by substrate-limited decomposition of litter and humus. A fraction of the decomposed C is lost by C mineralisation, another minor fraction is humified directly, and the rest is built into the microbes. Accumulation of C in microbes will result in a change in the C/N ratio. If the resulting microbial C/N ratio is higher than the N demand by microbes, this will lead to a net immobilisation; if it is lower than the N demand it will result in net mineralisation. A fraction of the microbial biomass is recycled into litter and humus (see Fig. 1). Microbes take up ammonium N, but in case of a shortage of N,

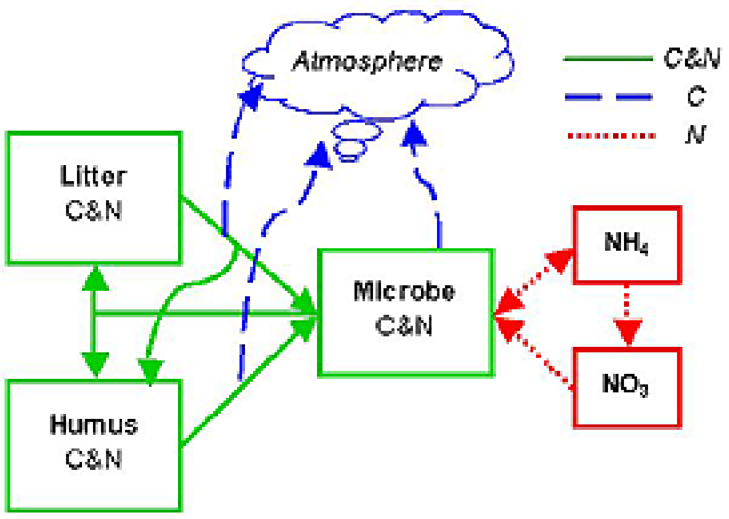

**FIGURE 1.** Pools of organic matter in each soil layer and the transfer and decomposition pathways for C and N into and between the C and N pools in the soil.

nitrate N is also taken up. In general, mineralisation of organic material leads to significant losses of C per molecule N taken up because of microbial respiration. On the other hand, the uptake of organic N with the low C/N ratio of amino acids is accompanied by a small soil C loss and will tend to increase the C pool and the C/N ratio in the soil. Further details are given elsewhere[9,10,11,27].

### Calibration of SOILN

#### *Driving Variables and Initial Values*

Driving variables for climate and soil water and temperature conditions, as well as soil physical characteristics, layer thickness, and root depth, were derived by simulations with a water and heat model (SOIL) for the site[21]. Daily deposition values were achieved by adjusting the N concentration of wet deposition to give a total deposition flux equal to the measured annual throughfall flux. Correspondingly, the fraction of ammonium N in input was put equal to the fraction in throughfall (52%). Initial biomass dry weight and N concentrations in various compartments were set in accordance with our measurements. Nitrification and denitrification were set very low according to measured values (<0.05 g $m^2$ $year^{-1}$)[12]. Finally, it was assumed that the root system and the microbes could take up 20% of the available mineral N pool within 1 day ($c_u$ in Eq. 3 above). This value was the same as used for a spruce stand at Sollingen in Germany[10] and more than twice as high as values usually used for agricultural crops[9,29]. The annual plant N uptake is insensitive to high values of the uptake fraction, since practically all N mineralised is taken up by plants. In case of shortage of N, transport between soil layers had the lowest priority. The deficit was covered by reducing the vertical soil-N flow by 50% of the deficit. Plant and microbial uptakes were reduced by 10% each of the deficit. This procedure was repeated within the time step until the whole deficit was covered. The calibration of SOILN was done stepwise, starting with the plant growth and litterfall and followed by the plant N and soil C and N dynamics.

***Step 1: Calibrating Photosynthetic Assimilation of Energy and Allocation of Biomass.*** The biomass N concentration was kept constant (as a boundary condition) by fixing it at the observed average value. Hence, N supply was not simulated during this calibration step. Potential radiation use efficiency and biomass allocation into stem, needles, and roots and the mortality of these pools were calibrated to obtain biomass production and pools in accordance with our measurements. Production of woody litter and leaf litter was fitted with help of their relative daily "mortality rates." We assumed that the canopy in this old stand did not expand during the modelling period, but stayed more or less constant, with only the stems growing[30]. The fraction of wood and canopy removed to litter was scaled individually each year to match the litterfall measurements.

***Step 2: Calibrating Plant N Uptake and C and N Dynamics in the Soil.*** The plant C and N dynamics and leaf-area development calibrated above was kept fixed as a boundary, and the soil C and N dynamics were calibrated. This calibration set the input of C and N to the soil organic pools, the mineralisation rates for those pools, the mineral N uptake, and the direct organic N uptake by mycorrhiza and the C/N ratio for the microbes. The microbial biomass was assumed to have a turnover time of once each half year and the microbial efficiency (g C retained in new microbial biomass per g C decomposed) was assumed to be 40% in the litter substrate[31] and 20% on the humic substrate[32]. The direct humification rate was set to 10% of the microbial C assimilation. The main criterion for the calibration was to supply sufficient N to the plant pools and at the same time keep the soil pools of N and C constant or slightly increasing, since the yearly decomposition rate is smaller than the litter input rate[12]. The adsorption capacity was adjusted by comparing the modelled and measured soil-solution concentrations of $NH_4$. Two different calibrations were done under this step: (1) a calibration with a traditional N-uptake approach where N is mineralised before uptake as mineral N; and (2) a calibration where N can be taken up both from the mineral pool and directly from the organic pool, as described above.

***Step 3: Final Model Tuning without Fixed Boundaries.*** The boundary conditions used in the previous steps were finally removed and all the calibrated parameters were adjusted according to the same criteria used in the previous steps (Table 3). Only minor corrections of steps 1 and 2 were needed.

***Step 4: Validation of SOILN***. Thereafter, the calibrated model was applied to the 3-year N-addition experiment without any change in calibration and model outputs were compared with measurements. The model was further validated by comparing model output with independent measurements of N-mineralisation and soil-solution concentrations of N during the 7 years of observation.

### RESULTS AND DISCUSSION

The model was calibrated to match model outputs for biomass production, biomass allocation, and N content in biomass with measurements and estimates (Fig. 2). The modelled growth rate is mainly determined by the photosynthetic assimilation of C and is restricted by the availability of N. The canopy was the most dynamic pool, responding rapidly to changes in N availability and litterfall.

The decomposition rate in the model was to a large extent determined by the climatic conditions. Consequently, mineralisation showed a strong seasonal dependency but also strong year-to-year differences as illustrated by the high mineralisation rates during the relatively "warm" winters of 1988 and 1989 (Fig. 3). On the other hand, severe droughts during the spring or summer resulted in strongly reduced mineralisation, as illustrated in the summers of 1989, 1992, and 1993 (Fig. 3). The increased mineralisation in 1988 to 1989, followed by reduced mineralisation in 1992 to 1993 (Fig. 3) clearly affected the organic pools of N (Fig. 4). The modelled mineralisation rate in the organic soil layer (top 0 to 7 cm) showed similar overall dynamics, both without and with the inclusion of organic N uptake, and the modelled dynamic correspond to the field observations (Fig. 3). The mineralisation rate in the calibration without organic N uptake is a factor 2 higher than measured (Fig. 3).

**TABLE 3**
**Main Parameter Settings in the Application SOILN Model to the Klosterhede Control Plot**

| | Ecosystem/Model Compartment | | | | |
|---|---|---|---|---|---|
| **Biomass Parameters** | **Unit** | **Leaf** | **Stem** | **Root** | **General** |
| **Plant growth** | | | | | |
| Radiation use efficiency | g DW MJ$^{-1}$ | | | | 1.2* |
| **Biomass allocation** | | | | | |
| Leaf area development | m$^2$ gDW$^{-1}$ | 0.00065 | | | |
| Root growth (fraction of total) | % day$^{-1}$ | | | 15 | |
| **N allocation** | | | | | |
| Minimum N level in needles | % | 0.5 | | | |
| Maximum N concentration | % | 2.0 | 0.15* | 0.63* | |
| **Litter production** | | | | | |
| Fraction of biomass | % day$^{-1}$ | 0.04* | 0.0006* | 0.03* | |

| | Ecosystem/Model Compartment | | | |
|---|---|---|---|---|
| **Soil Parameters** | **Unit** | **Litter** | **Humus** | **General** |
| **N uptake** | | | | |
| Mineral N uptake | % day$^{-1}$ | | | 20 |
| Organic N uptake (fraction of pool) | % day$^{-1}$ | 1 | 0.012* | |
| Plant N deficit at which organic N uptake is at maximum | g N m$^{-2}$ | | | 0.1 |
| **Mineralisation** | | | | |
| Gross decomp. (fraction of substrate) | % | 3.0 | 0.0047* | |
| Microbial efficiency (Net/gross rate) | | 0.4 | 0.2 | |
| Microbial mortality rate | % day$^{-1}$ | 0.4 | 0.2 | |

*Note:* Values achieved by calibration are denoted with an asterisk (*).

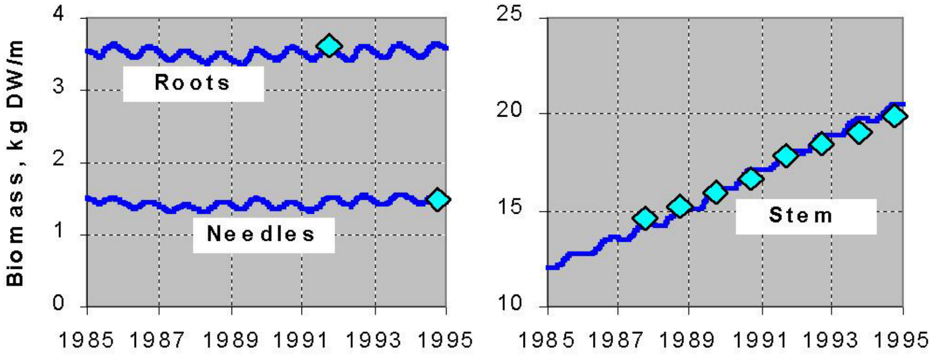

**FIGURE 2.** Simulated stem, root, and needle biomass development (lines)(kg DW m$^{-2}$) compared to estimated values based on field measurements (diamonds).

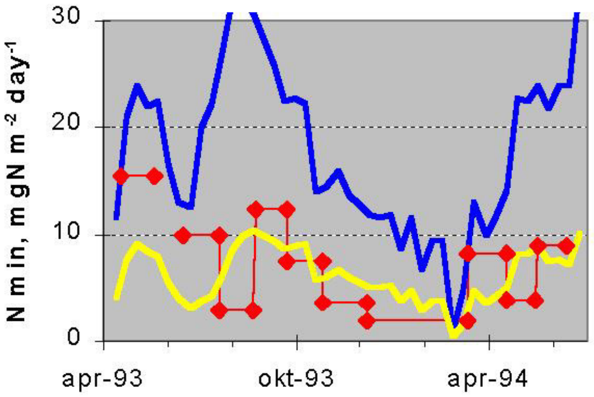

**FIGURE 3.** N mineralisation (mg N m$^{-2}$ day$^{-1}$) in the control plot at Klosterhede. Simulated mineralisation for the top 7 cm organic soil layer (1993 to 1994) without (dark line) and with (light line) organic N uptake compared with field measurements (thin line and diamonds).

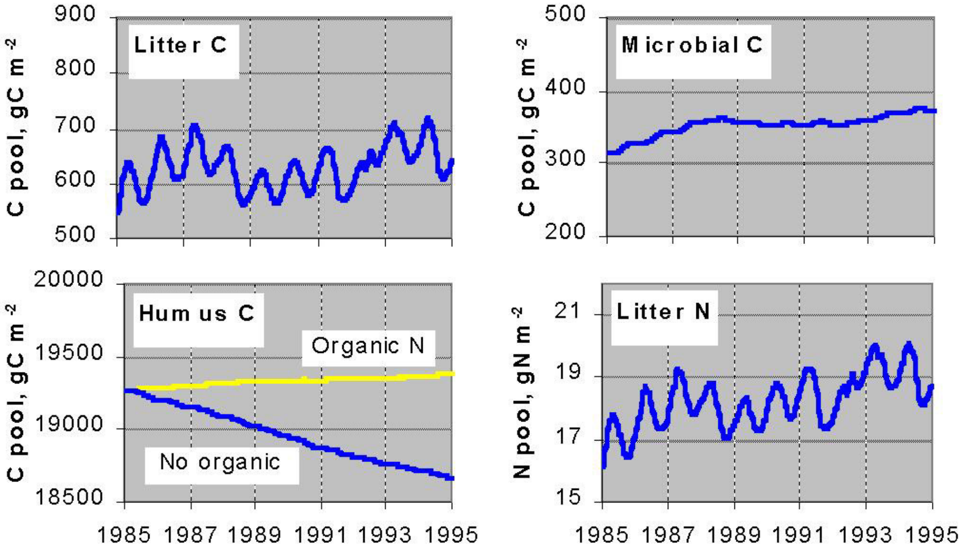

**FIGURE 4.** Simulated litter, microbial, and humus C and litter N development at Klosterhede, 1985 to 1995. Humus C also shows soil C pool for SOILN application with organic N uptake (light coloured line).

The litter, humus, and microbial C and N pools fluctuated over the years in accordance with fluctuations in input and mineralisation, but the net change over the 10-year period modelled was very small (Fig. 4). The litter and microbial pools received the litterfall directly and had the highest turnover rates. Consequently, those pools showed a substantial dynamic with seasonal as well as year-to-year fluctuations in C and N pools, and they were relatively unaffected by the two calibrations with and without organic N uptake. The more slowly developing humus pool showed only small fluctuations, but a clear difference in the dynamics of the C pool between the calibration including organic N uptake (C pools increase) and the calibration where N was only taken up as mineral N (C pools decrease) (Fig. 4).

Changes in the mineralisation rates affect the availability of N for the trees and, thereby, the potential for plant uptake and N status. Large fluctuations in the needle content of N were in fact seen, with modelled concentrations increasing until 1989 corresponding to the increase in mineralisation (Fig. 5). The concentration of N in the needles showed very little dependence on the two different N-uptake pathways. Despite a large mineralisation in 1989, the needle-N concentration decreased. This is because the increased mineralisation occurred during the winter when the plant uptake was small, and because N availability for tree growth was reduced in the growing season by immobilisation of C and N in the litter and microbial pools.

The needle-N concentrations were generally low at Klosterhede (1 to 1.5%) and the simulated needle-N concentration corresponded reasonably well with the observations, although the model underestimated the needle-N concentrations in the dry years from 1992 to 1994. The increasing needle-N concentra-

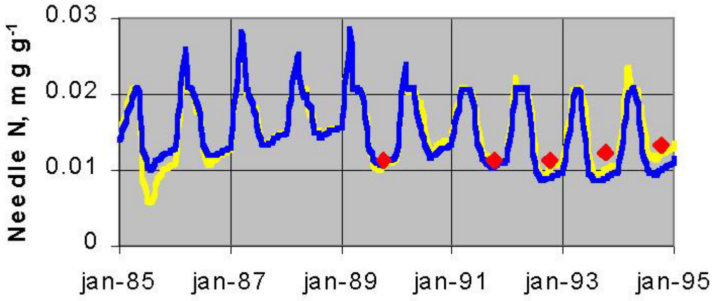

**FIGURE 5.** Simulated needle N concentration (mg N $gDW^{-1}$) in the control plot (1985 to 1995) without organic N uptake (dark line) and with organic N uptake (light coloured line) compared with measurements (diamonds).

tions observed from 1992 to 1994 were surprising since the N input did not change, but it may have been a drought effect, which is not explicitly included in the model. The springs of 1993 and 1994 were very dry and tree growth was strongly affected. Shoots were shortened and needle weight lower than normal but with higher N concentration[12].

In the model application including the alternative supply of N to the plants by direct uptake from the organic pool, this organic N supply was modelled to account for c. 35% of the total N uptake over the 10 years, while the remaining 65% was supplied by mineral N uptake (Fig. 6). Since the organic N uptake was assumed to compensate low mineral N uptake, the organic N uptake was highest in periods with low mineral N availability. This was illustrated by the period after 1989, when a larger fraction of mineralised N was taken up by microbes, causing less mineral N to be available for plant uptake. Consequently, plants took up more organic N, accounting for a larger proportion (>41%) in the latter part of the modelling period compared to c. 30% before 1989. In certain years as much as 46% of the total N uptake in the plants was supplied by organic uptake (Fig. 6).

The N concentrations in the soil solution were generally highest in the model application without organic N uptake because the mineralisation rate was highest in order to supply sufficient N to the plants. The modelled soil and soil-solution concentrations of $NO_3$ and $NH_4$ were in the same level as observed, although $NH_4$ concentrations seem to be overestimated during the winters in the calibration without organic N uptake (Fig. 7).

The leaching of N from the soil at Klosterhede is assumed to be very small, as indicated by low soil-solution concentrations. SOILN calculated an output of 0.27 and 0.45 g N $m^{-2}$ $year^{-1}$ in average over the 10 years with and without organic N uptake, respectively. This is somewhat higher than generally observed[12,21].

### Modelling the Effects of N Addition

The N addition was performed in quarterly or monthly doses and increased the N availability as indicated by both measured and modelled increases in N concentration in the soil solution (Fig. 8). The model reproduced N concentrations well with respect to timing as well as the N-concentration level, although $NH_4$ concentrations were somewhat overestimated when N had to be mineralised before uptake. Nitrate uptake or immobilisation at depths 25 to 55 cm was underestimated and resulted in exces-

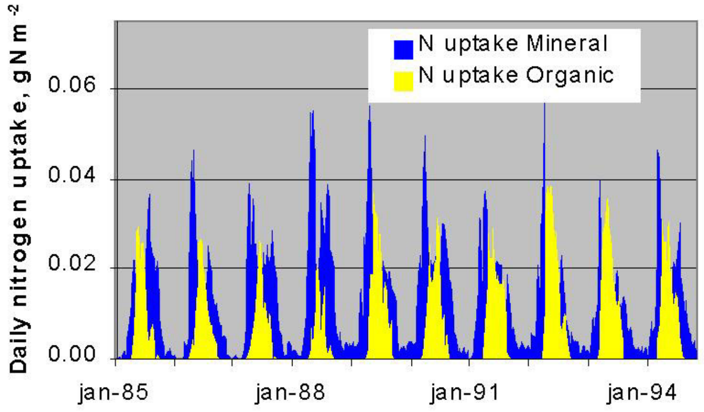

**FIGURE 6.** Simulated daily plant uptake of N from the mineralised (dark) and organic N pool (light) at Klosterhede, 1985 to 1995 (g N $m^{-2}$ $day^{-1}$).

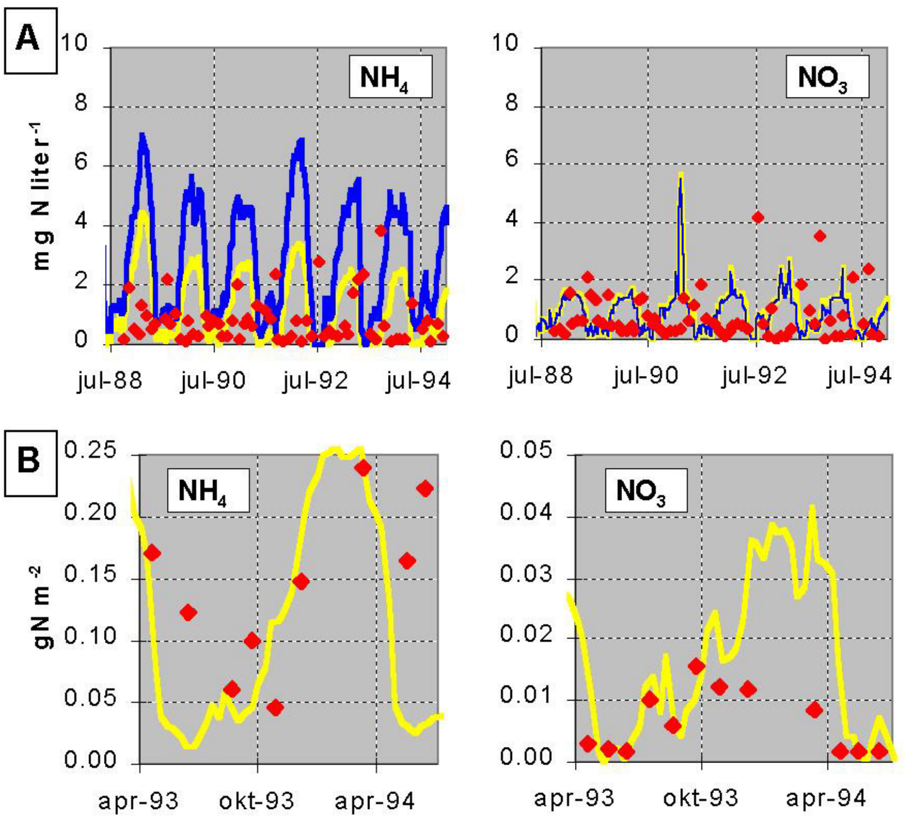

**FIGURE 7.** Comparison of simulated and measured $NH_4$ and $NO_3$ concentrations in (A) the soil water just below the top organic soil layer (mg N $l^{-1}$) without organic N uptake (dark line) and with organic N uptake (light line); and (B) the soil in the top 0- to 7-cm soil layer (g N $m^{-2}$) for the calibration with organic N uptake (light coloured lines); all compared with measurements (diamonds).

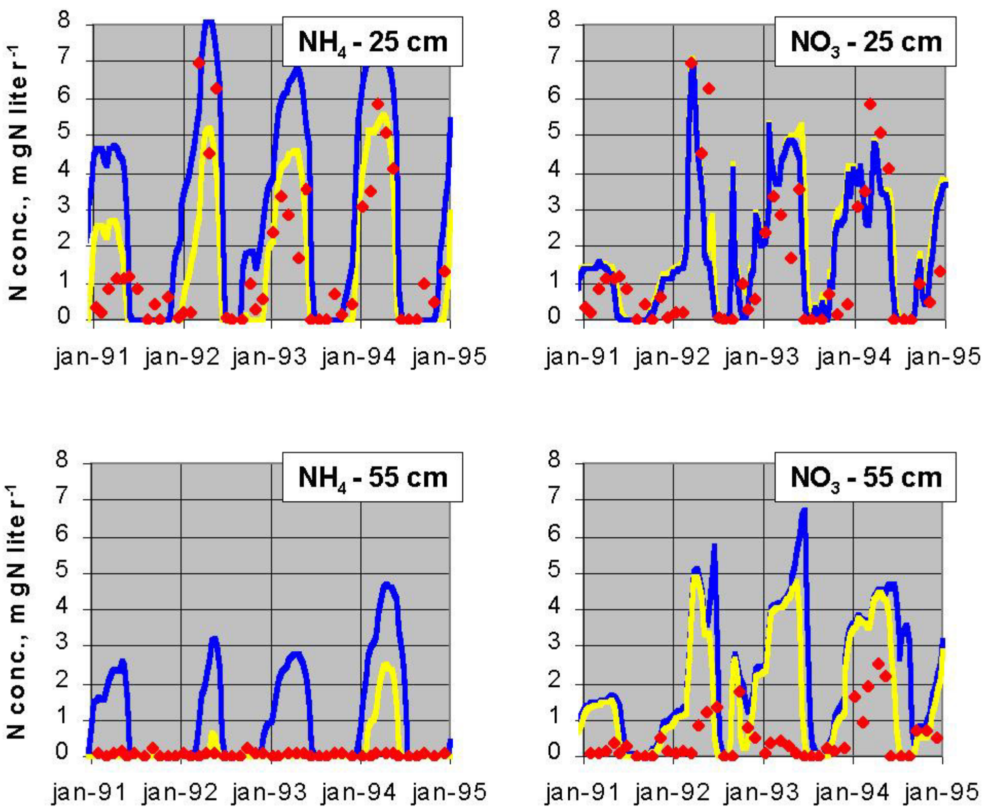

**FIGURE 8.** Simulated soil water concentrations of $NH_4$ and $NO_3$ (mg N $l^{-1}$) in different soil depths during N addition at Klosterhede without organic N uptake (dark line) and with organic N uptake (light coloured line) compared with measurements (diamonds).

sively high nitrate concentrations in the deep soil. The increased N availability immediately caused increased mineral N uptake in the growing season and a simultaneous reduction in the mycorrhizal N uptake in the application, including organic N uptake (Fig. 9). As a further consequence of the increased N availability, the N concentration in the needles increased by 0.003 g N $gDW^{-1}$ (Fig. 10), which agrees well with the observations.

The total addition of N for the period from 1992 to 1995 was 3.5 g N $m^{-2}$ $year^{-1}$ to the N-addition plot, in addition to the 1.9 g N $m^{-2}$ $year^{-1}$ supplied by deposition. The extra N input was distributed among the plants, soil, and leaching by the proportions 59, 21, and 21%, respectively, for the calibration without organic N uptake, and 35, 47, and 15%, respectively, including organic N uptake (Table 4). This distribution of N input predicted by the model corresponds reasonably well with field observations (54, 38, and 8%, respectively) based on the fate of $^{15}N$ performed in the beginning of the experiment (Table 4), although it reflects the general overestimation of the leaching losses. However, the numbers are not directly comparable as the $^{15}N$ measurements showed the distribution of the N that was added, while the model calculated the net distribution to each pool; that is, including N mineralised from organic pools. Leaching of N from the soil profile was estimated higher by the model compared to the measurements.

The increased availability of mineral N caused by the N addition increased the total plant N uptake by 19 and 31% both

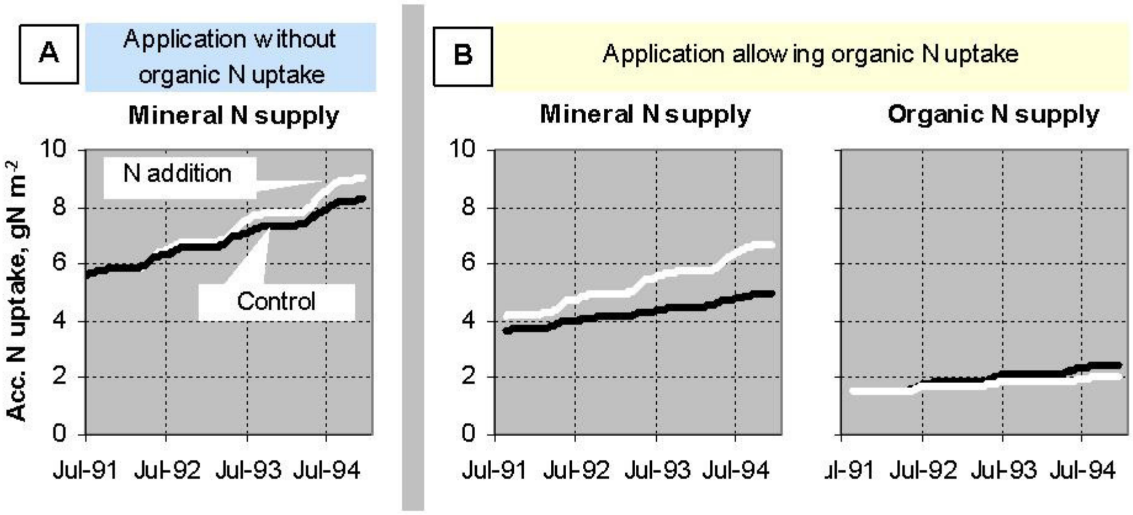

**FIGURE 9.** Simulated accumulated plant N uptake (g N $m^{-2}$) from the mineral and the organic N pool in the control (black lines) and N-addition (light lines) plots, 1991 to 1995. (A) Effect of N addition on mineral N uptake in model application without allowing direct uptake of organic N. (B) Effect of N addition on mineral and organic N uptake in model application allowing direct uptake of organic N.

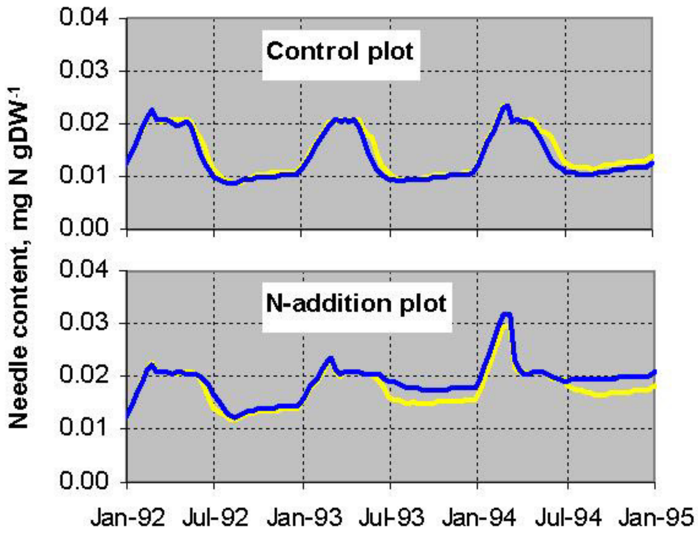

**FIGURE 10.** Simulated needle concentration of N (mg N $gDW^{-1}$) in the control and N-addition plots without organic N uptake (dark line) and with organic N uptake (light line), 1992 to 1995.

**TABLE 4**
**Modelled Input, Output, and Distribution of N in Control and N-Addition Plots at Klosterhede during the N-Addition Period 1992 to 1995**

| | | | Accumulation | | Uptake | | |
|---|---|---|---|---|---|---|---|
| | Input | Output | Plants | Soil | Mineral | Organic | % Organic |
| **Application without organic N uptake** | | | | | | | |
| Control (g N $m^{-2}$ $year^{-1}$) | 1.9 | 0.5 | 1.2 | 0.2 | 8.0 | 0 | 0 |
| N addition (g N $m^{-2}$ $year^{-1}$) | 5.3 | 1.2 | 3.2 | 0.9 | 10.5 | 0 | 0 |
| **Application including organic N uptake** | | | | | | | |
| Control (g N $m^{-2}$ $year^{-1}$) | 1.9 | 0.3 | 1.4 | 0.3 | 5.3 | 2.8 | 35 |
| N addition (g N $m^{-2}$ $year^{-1}$) | 5.3 | 0.8 | 2.6 | 1.9 | 8.1 | 1.5 | 16 |
| **Distribution of added N in N-addition plot** | | | | | | | |
| Without organic N uptake (%) | | 21 | 59 | 21 | | | |
| With organic N uptake (%) | | 15 | 35 | 47 | | | |
| Measured by $^{15}N$ (%) | | 8 | 54 | 38 | | | |

*Note:* Distribution of added N inferred from the fate of $^{15}N$ was only based on data from 1 year (1992 to 1993)[12].

with and without organic N uptake, respectively. The increased N uptake was not allocated to increased growth but to increased N status of the different plant parts[12]. In the application including organic N uptake, the increase in N uptake was supplied by mineral N, which increased by 53%, whereas organic uptake was reduced by 46% compared with simulations for the control plot (no N addition). So, the model results indicate that during the 3 years of N addition, the organic uptake was reduced from supplying 35 to 16% of the plant N uptake (Table 4). These results cannot be validated directly from measurements. However, recent findings of a significant reduction in mycorrhizal fruit bodies following fertilisation[33] and N addition[34] also suggest a decreasing activity of mycorrhiza.

Following the N addition, the simulated net mineralisation of N increased slightly (c. 10% during summer; constant during winter). On the other hand, the field measurements showed increased net mineralisation of up to 80%[12]. However, this increased net mineralisation was hypothesised to result from a decrease in immobilisation rather than an increase in mineralization[12], which cannot be checked by this model.

The total input of c. 5.3 g N $m^{-2}$ $year^{-1}$ during the N-addition period strongly exceeds the demand for the stand and the critical threshold of c. 2.5 g N $m^{-2}$ $year^{-1}$[35]. It may therefore be expected that a higher fraction of the input would be leached to the groundwater. However, as recently described[3], not only the N load itself but also the N status of the soil is of major importance. It has been indicated that the N status may be accurately reflected by the C/N in the forest floor, and that C/N 25 may serve as an approximate threshold below which increased N leaching is likely to appear[3]. This means that leaching of significant amounts of the input is not to be expected at the C/N ratio at Klosterhede c. 32 and a large part of the extra input is incorporated into the soil organic layer, causing the C/N to decrease. In the long run the accumulation in the soil will cause the N status to increase and increase the risk for N leaching. This incorporation of the N into the soil organic matter is well described by the model. On the other hand, the mechanism responsible for the change in the fate of the N input as the N status increases (and C/N in the forest floor decreases) is not well known and is currently not described in the model.

### Evaluation of SOILN

The experiment at Klosterhede included a control plot and an N-addition plot. Calibration to the control plot and subsequent application to the N-addition experiment tested the SOILN model. The forest at Klosterhede is characterised by a low N status reflected in low N concentrations in the needles and a high C/N ratio in the soil. This situation and the increased accumulation of N in the tree biomass as a response to N addition are both well reproduced in the calibration of the model. Also, the overall distribution of added N among soil, plants, and leaching is reasonably well reproduced. However, details in the seasonal pattern in the N cycle at Klosterhede, mainly linked to mineralisation during winter and drought, were not well reproduced and need further improvement.

There are still uncertainties in many of the parameters and measurements, especially concerning the soil pools of C and N and the mineralisation, which is at the same time the most sensitive part in the model. This may of course limit the accuracy of the model output, especially as regards the details in the internal distribution of C and N in and between the different soil layers. On the other hand, the combination of N-load regimes and N-pool responses strongly constraints the model and limits the flexibility in the output. In the present application, small errors in the distribution of C and N among the layers and organic pools are of minor importance because the changes in pools sizes are small and have little influence on the output. In case we want to test a “non-steady state” change such as the long-term response to climate change with increased mineralisation, the estimation of the various pool sizes and soil layers is absolutely crucial.

### CONCLUSION

Application of the SOILN model to Klosterhede generally matched the observations well, independent of the simulated pathway for N uptake. However, the simulations without the organic N uptake module resulted in high mineralisation rates for the organic pools and therefore, these pools were depleted. Furthermore, the increased mineralisation rate lead to an increased concentration of $NH_4$ in the soil solution and increased leaching of N at a level exceeding the observations in the field. The inclusion of a pathway for direct N uptake from the organic soil pools to the trees improved the output. The organic N uptake accounted for a significant fraction of the total N uptake, being 33% in average over 10 years and in single years accounting for as much as 46%. These numbers are calculated on the assumption that the direct canopy uptake of N from the atmosphere was negligible. If a direct canopy uptake of atmospheric N occurs, this will reduce the need for organic N uptake in the roots correspondingly. Data from Klosterhede have indicated that a small uptake of N in the canopy may occur[36].

Inclusion of a pathway for organic N uptake changed the overall C and N balance of the ecosystem because the mineralisation rates were lower. This reduced the mineral N content in the soil and soil solution and it reduced the C loss from the organic soil pools by c. 100 kg C $ha^{-1}$ $year^{-1}$ over the 10-year period tested. Quantitatively, this difference in C storage among the two strategies is small (c. 1% of total C content in the top soil layer per year), and in most cases it will be too small to be measured over time scales less than 10 to 25 years. However, this study illustrates how important N can be as "tracer" for changes in C dynamics, because C and N are closely linked and because N is often more sensitive to changes in biogeochemical cycling. It has to be noted that the direct uptake of organic N by transfer of amino acids should still be regarded as a working hypothesis, as the present study includes no measurements to verify the organic uptake specifically. Other mechanisms that would also reduce the C loss, such as fast mineralisation of specific low C/N compounds, may exist. However, mycorrhizal association with plants, and the ability of plants to take up organic C and N, was demonstrated in several studies and it has been shown that amino acids may be a likely mode of transfer[14,16,17]. Furthermore, a justification that this pathway may account for significant amounts of N transfer was recently showed experimentally in field studies in Sweden[17] and in the arctic tundra[13]. However, the mechanism and the regulation of the transfer is still largely unknown. SOILN describes organic N uptake to be regulated by the N status of the plants and the availability of inorganic N in the soil. This expression gave a good agreement with experimental results for the overall N dynamics in the soil and plants. However, our findings underline the need for further experimental work to verify and quantify the pathway and possible mechanisms for and regulation of organic N uptake.

### ACKNOWLEDGEMENTS

The work was financially supported by SNS (the coordinative board for Nordic forest research), the Danish Agricultural Ministry, RISØ National Laboratory, Swedish University of Agricultural Sciences, and Danish Forest and Landscape Research Institute. We are grateful to the many people who were involved in the extensive field and laboratory work. The work was conducted within the Nordic nitrogen project, NORN, and we want to thank the participants in NORN for inspiring and fruitful discussions.

| | | |
|---|---|---|
| Received: | July | 10, 2001 |
| Revised: | November | 12, 2001 |
| Accepted: | November | 13, 2001 |
| Published: | December | 12, 2001 |
